# Infant feeding knowledge, attitudes and practices of HIV-positive breastfeeding mothers

**DOI:** 10.4102/hsag.v29i0.2617

**Published:** 2024-07-31

**Authors:** Kgabo M. Mabotja, Annette van Onselen, Reno E. Gordon

**Affiliations:** 1 Department of Human Nutrition & Dietetics, School of Health Care Sciences, Sefako Makgatho Health Sciences University, Pretoria, South Africa; 2 Department of Life and Consumer Sciences, College of Agriculture and Environmental Sciences, University of South Africa, Johannesburg, South Africa

**Keywords:** infant feeding, HIV-positive mothers, exclusive breastfeeding, mixed feeding, replacement feeding

## Abstract

**Background:**

Assessment of infant feeding knowledge, attitudes and practices of human immunodeficiency virus (HIV)-positive breastfeeding mothers may determine compliance with the chosen feeding method.

**Aim:**

The study assessed knowledge, attitudes and practices on infant feeding among HIV-positive breastfeeding mothers.

**Setting:**

The study was conducted at five clinics in the Chief Albert Luthuli sub-district of Mpumalanga, South Africa.

**Methods:**

A descriptive cross-sectional study with a convenient sample of 155 HIV-positive breastfeeding mothers.

**Results:**

More than half of the participants (54.8%) were knowledgeable of exclusive breastfeeding in general. However, less than half were knowledgeable of exclusive breastfeeding in the context of HIV (46.5%), mixed feeding (28.4%) and replacement feeding (49.0%). Most participants (85.8%) reported that they were advised to exclusively breastfeed for 6 months, 61.3% intended to exclusively breastfeed for 6 months, and 29% intended to stop breastfeeding at 6 months. Most participants (64.5%) intended to introduce solids at 6 months, and for participants who intended to introduce solids before 6 months, 37.7% did not believe that exclusive breastfeeding was sufficient for the baby.

**Conclusion:**

Although most participants were knowledgeable about exclusive breastfeeding, there were misconceptions that required attention such as the lack of knowledge on exclusive breastfeeding in the context of HIV, mixed feeding and replacement feeding. Exclusive breastfeeding for 6 months was the most emphasised infant feeding practice.

**Contribution:**

This study builds on existing literature on infant feeding knowledge, attitudes and practices and provides a basis for interventions for improved exclusive breastfeeding rates.

## Introduction

Breastfeeding is the optimal way to provide adequate nutrition for an infant. The type (exclusive breastfeeding, mixed feeding or formula feeding) and the duration of feeding that the mother chooses determine the risk of vertical transmission of human immunodeficiency virus (HIV) in HIV-exposed children (Young et al. 2011). Exclusive breastfeeding by HIV-positive mothers, although not totally risk-free regarding HIV transmission, can contribute to the prevention of non-HIV morbidity and infant HIV-free survival (WHO 2016). Therefore, breastfeeding is recommended for 2 years or more while ensuring maternal antiretroviral treatment (ART) use (South African National Department of Health 2019).

Human immunodeficiency virus-exposed infants who are not exclusively breastfed are at a greater risk for contracting HIV because the supplementary food given before 6 months damages the intestinal lining (Nankumbi & Muliira 2015). The prevention of other diseases in infants has also been associated with appropriate breastfeeding practices. For example, exclusive breastfeeding is associated with fewer cases of diarrhoea in both HIV-exposed infants and infants born to HIV-negative mothers (Rollins et al. 2013). The risk of morbidity and mortality associated with diarrhoeal diseases is also reduced with exclusive breastfeeding (Lamberti et al. 2011).

South Africa has a history of low exclusive breastfeeding rates among infants younger than 6 months of age (WHO & UNICEF 2019). Human immunodeficiency virus-infected mothers are reluctant to breastfeed their infants for fear of infecting them with HIV (Goga et al. 2012). In 2016–2017, there was a 35.3% decrease in the exclusive breastfeeding rate in Mpumalanga when compared to 2015–2016 on the district health barometer. The Gert Sibande district in Mpumalanga was noted to be one of the districts with the lowest exclusive breastfeeding rates (24.9%) in South Africa (Massyn et al. 2017).

It is important to determine the knowledge, attitude and practices on infant feeding among HIV-positive breastfeeding mothers as it determines compliance with the chosen feeding method. Mothers who are knowledgeable may make informed choices regarding infant feeding practices. Furthermore, it will also identify gaps that need to be filled by health care professionals who are responsible for counselling mothers on infant feeding both at the clinic and hospital levels. Mothers should be educated on all infant feeding options so that when they decide on an infant feeding option, they do so in a manner that is safe and limits the risk of mother-to-child transmission of HIV.

This study is important as in the Chief Albert Luthuli sub-district, only 0.3% of infant birth polymerase chain reaction (PCR) were positive in 2019–2020. However, positive infant PCR at 10 weeks increased to 14.5%, and only 22.3% of infants are exclusively breastfed up to 6 months (Gert-Sibande district health plan 2018/2019-2020/2021). Delayed testing and introduction of treatment could also be a reason for the increase in the positive infant PCR at 10 weeks (Lilian et al. 2012). It is important to establish the views on infant feeding of mothers because it provides an idea of what their infant feeding practices will be like after discharge. The study is important as it could help provide information that could inform future interventions to reduce the rate of mother-to-child transmission of HIV.

## Research methods and design

### Study design

A quantitative descriptive study was used to assess knowledge, attitudes and practices on infant feeding among HIV-positive breastfeeding mothers at clinics around Chief Albert Luthuli sub-district of Mpumalanga from November 2022 to April 2023.

### Study population and sampling strategy

The study population was 1030 HIV-positive breastfeeding mothers attending growth monitoring appointments in the Chief Albert Luthuli sub-district. There are 21 clinics in the Chief Albert Luthuli sub-district, and systematic sampling was used to select the clinics included in the study. All clinics in the Chief Albert Luthuli sub-district were listed in alphabetical order, and grouped as Northern and Southern clinics. Every fourth clinic was selected and included in the study amounting to five clinics being included in the sample. Therefore, the following clinics were included: Kroomdraai clinic, Nhlazatshe 4 clinic, Bettysgoed clinic, Fernie 1 clinic and Silobela clinic. Non-probability convenience sampling was used to select the participants to be included in the study. A sample size of 155 was calculated based on the population size of 1030 using the online Raosoft sample size calculator with a 5% margin of error, 95% confidence level and 50% response distribution, with 31 participants from each of the five clinics included.

### Data collection

Participants were interviewed using a questionnaire based on the 2010 World Health Organization (WHO) infant feeding guidelines and the 2016 updated guidelines on HIV and infant feeding (WHO 2010, 2016). The questionnaire had three sections assessing knowledge, attitudes and practices and was translated into siSwati because the majority of the participants were siSwati speaking.

### Reliability

The study methods were applied consistently, and the procedures were standardised by conducting training before the commencement of data collection. The questionnaire was piloted.

### Validity

The questionnaire was developed based on the 2010 WHO infant feeding guidelines and the 2016 updated guidelines on HIV and infant feeding (WHO 2010, 2016).

### Data analysis

The questionnaire data were coded and entered into a Microsoft Excel spreadsheet and imported to the Statistical Package of Social Sciences (SPSS) version 29 for analysis. Descriptive statistics were performed for frequencies and percentages of the knowledge, attitudes and practices data. If participants answered 60% or more of the knowledge questions correctly, they were categorised as knowledgeable. Those who answered less than 60% of the knowledge questions correctly were considered to have poor knowledge. This is similar to a recent study assessing the breastfeeding knowledge of mothers in Limpopo, South Africa which also used 60% as the benchmark for adequate knowledge (Motadi, Malise & Moshaphi 2019). Attitude responses were based on the Likert agreement scale which includes options such as strongly disagree, disagree, neutral, agree and strongly agree.

### Ethical considerations

All participants gave informed consent before participating in the study which was conducted per the Declaration of Helsinki, and the ethical clearance was approved by the Sefako Makgatho Health Sciences University Research Ethics Committee (SMUREC/H/41/2022).

## Results

### Sociodemographic characteristics

Most of the participants (45.8%) were 26–35 years of age, spoke siSwati (86.5%), completed matric (42.6%), were unemployed (80.6%), single (83.2%) and had two children (45.2%).

### Infant feeding knowledge

As presented in Table 1, more than half (54.8%) of the participants were knowledgeable (knowledge score > 60%) of exclusive breastfeeding in general. More than half (53.5% and 51.0%) were not knowledgeable (knowledge score < 60%) about exclusive breastfeeding in the context of HIV and replacement feeding. In addition, more than two-thirds of participants (71.6%) were not knowledgeable about mixed feeding.

**TABLE 1:** Summary of infant feeding knowledge.

Infant feeding variable	Knowledgeable		Not knowledgeable	
	*n* = 155	%	*n* = 155	%
Exclusive breastfeeding in general	85	54.8	70	45.2
Exclusive breastfeeding in the context of HIV	72	46.5	83	53.5
Mixed feeding	44	28.4	111	71.6
Replacement feeding	76	49.0	79	51.0

HIV, human immunodeficiency virus.

Table 2 indicates the participants’ responses to statements regarding knowledge of infant feeding. The correct responses are indicated in *italics*, and the responses of the majority of participants are indicated in bold. In cases where the responses of the majority of participants were correct, these are indicated in bold and italics. Therefore, for knowledge on exclusive breastfeeding not specific to HIV, most participants provided correct responses to nine of the 15 statements, equating to 60% correct answers. Regarding knowledge of breastfeeding in the context of HIV, the majority of participants provided correct answers to five of the six statements regarding mixed feeding, equating to 83% correct answers. Most participants provided correct responses to six of the seven statements regarding mixed feeding, equating to 86% correct answers. Lastly, regarding knowledge of replacement feeding, the majority of participants provided correct responses to five of the six statements, equating to 83% correct answers.

**TABLE 2:** Detailed infant feeding knowledge.

Statement	True		False		Unsure	
	*n*	%	*n*	%	*n*	%
**Knowledge of exclusive breastfeeding in general (not specific to HIV)**						
Exclusive breastfeeding is not safe for the baby	**127**	**81.9**	*24*	*15.5*	4	2.6
Placing the baby on the mother’s chest helps with breast milk production	***70***	***45.2***	43	27.7	42	27.1
Exclusive breastfeeding causes malnutrition	**122**	**78.7**	*26*	*16.8*	7	4.5
Breastfeeding protects the baby from illnesses such as diarrhoea	***108***	***69.7***	23	14.8	24	15.5
Exclusive breastfeeding saves money	***135***	***87.1***	17	11	3	1.9
Exclusive breastfeeding helps prevent early unwanted pregnancy	***86***	***55.5***	37	23.9	32	20.6
The first milk from the breast should be thrown away	**93**	**60**	*26*	*16.8*	36	23.2
An exclusively breastfed baby under 6 months old should not drink water	***66***	***42.3***	61	39.4	28	18.1
An exclusively breastfed baby should be fed four times per day	**94**	**60.6**	*32*	*20.7*	29	18.7
Breastfeeding helps mother and baby to bond	***124***	***80***	17	11	14	9.0
The mother should breastfeed her baby 2 h after delivery	49	31.6	***53***	***34.2***	53	34.2
Exclusive breastfeeding does not reduce the risk of jaundice in babies	57	36.8	*32*	*20.6*	**66**	**42.6**
When giving expressed breast milk to an exclusively breastfed baby, bottle-feeding is better than cup-feeding	**70**	**45.2**	*68*	*43.9*	17	11
The breast should be cleaned before each feed	40	25.8	***80***	***51.6***	35	22.6
Breast milk can be stored in the fridge when the mother goes to work	***79***	***51***	50	32.3	26	16.8
**Knowledge of breastfeeding in the context of HIV**						
Baby can get HIV through breast milk	***116***	***74.8***	14	9.0	25	16.1
It is important to use a condom during intercourse when breastfeeding	***120***	***77.4***	25	16.1	10	6.5
Taking antiretroviral drugs protects your baby from being infected with HIV when you are breastfeeding	***125***	***80.6***	11	7.1	19	12.3
When the mother is HIV-positive, breastfeeding should stop at 6 months	36	23.2	***90***	***58.1***	29	18.7
HIV-positive mothers can breastfeed their babies until 2 years	*54*	*34.8*	43	27.7	**58**	**37.4**
When one breast has an infection, the mother must stop breastfeeding from both breasts	54	34.8	*34*	*21.9*	**67**	**43.2**
**Knowledge of mixed feeding**						
Mixed feeding includes giving the baby water	***79***	***51***	42	27.1	34	21.9
Mixed feeding is safe when the mother is HIV-positive	**80**	**51.6**	*41*	*26.5*	34	21.9
A baby can get HIV when mixed-fed	***90***	***58.1***	32	20.6	33	21.2
Practising mixed feeding reduces the breast milk supply in breastfeeding mothers	***57***	***36.8***	51	32.9	47	30.3
From 3 months, baby can be given both solids and breast milk	63	40.6	***65***	***41.9***	27	17.4
Mixed feeding causes the baby to gain sufficient weight	**67**	**43.2**	*48*	*31*	40	25.8
Mixed feeding can cause breast problems in breastfeeding mothers	***64***	***41.3***	43	27.7	48	31
**Knowledge of replacement feeding**						
Formula milk is the same as breast milk	**92**	**59.4**	*47*	*30.3*	16	10.3
Formula milk protects babies from illnesses	**80**	**51.6**	*60*	*38.7*	15	9.7
Formula milk may be contaminated by manufacturing error	***92***	***59.4***	28	18.1	35	22.6
Formula milk may contain unsafe ingredients for the baby	***87***	***56.1***	29	18.7	39	25.2
Error in the preparation of formula milk can make the baby ill	***106***	***68.4***	22	14.2	27	17.4
Formula feeding exposes the baby to possible allergens and intolerances	***78***	***50.3***	30	19.4	46	29.7

Note: The responses of the majority of participants are indicated in bold and in cases where the responses of the majority of participants were correct, these are indicated in bold and italics.

HIV, human immunodeficiency virus.

### Infant feeding attitudes

Table 3 indicates the responses of the participants on attitude questions relating to infant feeding. The majority of participants (77.4%) agreed and strongly agreed that breastfeeding is an enjoyable experience. Less than half (45.2%) disagreed and strongly disagreed that breastfeeding is not appropriate in the 21st century. Less than half of the participants (47.4%) also disagreed and strongly disagreed that breastfeeding should not be practised, and 62.6% agreed and strongly agreed that breastfeeding is a special time for the mother and baby to bond. Most of the participants disagreed and strongly disagreed that breastfeeding is gross (72.3%) and tiring (67.1%).

**TABLE 3:** Attitude on infant feeding.

Statement	Strongly disagree		Disagree		Neutral		Agree		Strongly agree	
	*n*	%	*n*	%	*n*	%	*n*	%	*n*	%
**Attitude to exclusive breastfeeding**										
Breastfeeding is an enjoyable experience	10	6.5	8	5.2	17	11	**74**	**47.7**	**46**	**29.7**
Breastfeeding is not appropriate for the 21st century	**13**	**8.4**	**57**	**36.8**	73	47.1	10	6.5	2	1.3
If possible, breastfeeding should not be practised	**15**	**9.7**	**58**	**37.7**	57	37.0	21	13.6	3	1.9
Breastfeeding is a special time to spend with your baby	3	1.9	10	6.5	45	29.0	**72**	**46.5**	**25**	**16.1**
Breastfeeding is not adequate to help your baby grow	**31**	**20**	**34**	**21.9**	54	34.8	31	20	5	3.22
Breastfeeding is too time-consuming	**40**	**25.8**	**68**	**43.9**	15	9.7	23	14.8	9	5.8
Breastfeeding is gross	**42**	**27.1**	**70**	**45.2**	16	10.3	19	12.3	8	5.2
Breastfeeding is too tiring	**43**	**27.7**	**61**	**39.4**	20	12.9	23	14.8	8	5.2
**Attitude on mixed feeding**										
Mixed feeding is necessary to satisfy the baby	**24**	**15.4**	**41**	**26.5**	30	19.4	48	31	12	7.7
Mixed feeding should be practised so that the baby will thrive	**12**	**7.7**	**52**	**33.5**	40	25.8	41	26.5	10	6.5
If possible, mixed feeding should be practised	**13**	**8.4**	**40**	25.8	52	33.5	39	25.2	11	7.1
Mixed feeding should be practised so that the baby sleeps well at night	12	7.8	38	24.7	40	26	**56**	**36.4**	**8**	**5.2**
Mixed feeding is a waste of time and money	3	1.9	22	14.2	54	34.8	**68**	**43.9**	**8**	**5.2**
**Attitude to replacement feeding**										
I would choose to use formula milk rather than breast milk to feed my baby	**43**	**27.7**	**41**	**26.7**	12	7.7	36	23.2	23	14.8
Mothers should use formula milk only if they are unable to breastfeed	6	3.8	23	14.8	79	51	**42**	**27.1**	**5**	**3.2**
There is too much risk of intolerances and allergic reactions when using formula milk	6	3.8	21	13.5	64	41.3	**57**	**36.8**	**7**	**4.5**
I would stop breastfeeding if the hospital or clinic provided free formula	**42**	**27.1**	**49**	**31.6**	22	14.2	11	7.1	31	20

Note: The responses of the majority of participants are indicated in bold.

Regarding the participants’ attitudes towards mixed feeding, less than half of the participants (41.9%) disagreed and strongly disagreed that mixed feeding is necessary to satisfy the baby, and 41.2% disagreed and strongly disagreed that mixed feeding should be practised to help the baby thrive. More than a third of the participants (34.3%) disagreed and strongly disagreed that mixed feeding should be practised, 41.6% agreed and strongly agreed that mixed feeding should be practised to help the baby sleep at night, and 49.1% agreed and strongly disagreed that mixed feeding is a waste of time and money.

Lastly, regarding the participants’ attitudes towards replacement feeding, more than half of the participants (54.4%) disagreed and strongly disagreed that they would choose formula over breastmilk, and 30.2% agreed and strongly agreed that mothers must only use formula when they are unable to breastfeed. Less than half of the participants (41.3%) agreed and strongly agreed that there is too much intolerance and allergic reaction when using formula milk, and 58.7% disagreed and strongly disagreed that they would stop breastfeeding if offered free formula at the hospital or clinic.

### Infant feeding practices

Table 4 indicates the infant feeding practices of the HIV-positive breastfeeding mothers in the current study. Most participants (85.8%) reported that they were advised to exclusively breastfeed for up to 6 months, 61.3% intended to exclusively breastfeed for 6 months, and 29% intended to stop breastfeeding at 6 months. Less than two-thirds of the participants (64.5%) intended to introduce solids at 6 months, and for the participants who will introduce solids before 6 months, 37.7% did not believe that exclusive breastfeeding was not enough for the baby.

**TABLE 4:** Infant feeding practices.

Age of the infant	*N* = 155	%
**Duration advised to exclusively breastfeed**		
Up to 1 month	1	0.6
Up to 6 weeks	1	0.6
Up to 3 months	5	3.2
Up to 6 months	133	85.8
More than 6 months	15	9.7
**Intended breastfeeding duration**		
Up to 1 month	9	5.8
Up to 6 weeks	8	5.1
Up to 3 months	23	14.8
Up to 4 months	13	8.4
Up to 6 months	95	61.3
More than 6 months	7	4.5
**Intended time to stop breastfeeding**		
1 month	4	2.6
2 months	3	1.3
3 months	5	3.2
4 months	4	2.6
6 months	31	20
7 months	1	0.6
8 months	1	0.6
9 months	2	1.3
10 months	2	1.3
11 months	1	0.6
12 months	45	29
17 months	3	1.9
18 months	19	12.3
24 months	32	20.6
36 months	2	1.3
**Intended time to introduce solid foods**		
1 month	14	9
2 months	3	1.9
3 months	22	14.2
4 months	13	8.4
5 months	3	1.9
6 months	100	64.5
**Reason for introducing solids before 6 months**		
Response	*n* = 53	%
I am going to work	9	17
I am going to school	12	22.6
I want to have more time to myself	11	20.7
I don’t believe that breastfeeding alone will make the baby grow well	20	37.7
I believe it’s still called mixed-feeding	1	1.9

## Discussion

The current study assessed the knowledge, attitudes and practices of 155 HIV-positive breastfeeding mothers attending immunisation services at clinics in the Chief Albert Luthuli sub-district of Mpumalanga.

### Knowledge of exclusive breastfeeding in general

More than half of the participants in the current study were knowledgeable about exclusive breastfeeding in general. However, a concerning finding was the misconception regarding exclusive breastfeeding as more than 75% of mothers stated that exclusive breastfeeding is not safe for the baby, and it causes malnutrition. Similarly, in the Limpopo province of South Africa, Motadi et al. (2019) reported that 75% of mothers of infants younger than 2 years of age reported that breastfeeding should not continue for 2 years. These misconceptions can negatively affect the infant feeding practices of mothers and could explain the fact that 61% of participants in the current study indicated that they intended to breastfeed for 6 months only. This is contrary to the WHO and UNICEF recommendation of continued breastfeeding for up to 2 years or beyond (UNICEF 2018).

In the current study, 60% of participants stated that colostrum should be discarded. This is much greater than the 8.8% of first-time mothers in Northwest Ethiopia who discarded colostrum (Ayalew & Asmare 2021). This is a concern as the benefits of colostrum are well-documented such as enhancing immunity, among others, and discarding colostrum increases the risk of infections (El-Loly 2022; Lyons et al. 2020).

Half of the participants in the current study (51%) indicated that breast milk can be stored in the fridge when the mother goes to work or school. These findings are lower than the 81.5% of HIV-positive mothers in northeast Namibia who knew that when mothers go to work, they can continue exclusive breastfeeding by expressing their breast milk (Ashipala, Shikukumwa & Joel 2021). The participants in the current study might be at risk of mixed feeding their infants or stopping breastfeeding when they return to work or school because of their poor knowledge about the expression and storage of breast milk.

### Knowledge of exclusive breastfeeding (in the context of HIV)

Less than half of the participants (46.5%) were knowledgeable about exclusive breastfeeding in the context of HIV. In addition, 20% of the participants did not know that ART can protect their baby from being infected with HIV when breastfeeding. This is a concern as it is well-documented that ART can reduce HIV transmission among HIV-positive breastfeeding mothers (WHO 2023).

Most participants (74.8%) stated that a baby can get HIV through breast milk, which is similar to the findings of a study conducted in Cameroon that found that 89.1% of pregnant women knew that a baby could get HIV through breastfeeding (Sama et al. 2017). More than a third of the pregnant women in Mexico (35.7%) knew that HIV can be transmitted through breastfeeding (Becka et al. 2015), and 38.9% of pregnant women in Ethiopia responded that HIV could be transmitted from the infected mother to her baby through breastfeeding (Yeshaneh et al. 2023). A concerning finding in the current study was that one in five participants did not know that ART can protect an infant from being infected with HIV during breastfeeding. Furthermore, one in four participants did not know that a baby can get HIV through breast milk. Such a lack of knowledge increases the risk of HIV transmission from mother to child and highlights the need for health professionals to educate HIV-positive mothers on the importance of appropriate infant feeding practices coupled with the use of ART.

A concern is that more than half of the participants (58.1%) in the current study stated that when the mother is HIV-positive, breastfeeding should stop at 6 months, and 34.8% of the participants indicated that HIV-positive mothers can breastfeed their babies until 2 years of age. The WHO recommends that HIV-positive breastfeeding mothers exclusively breastfeed their infants for 6 months with the introduction of solid foods after 6 months and continue breastfeeding for up to 2 years and beyond (WHO 2016). A third of the mothers (34%) in the current study were aware that they could breastfeed for up to 2 years. This lack of knowledge could lead to low breastfeeding rates, resulting in infants not getting all the benefits of breastfeeding for longer.

### Knowledge of mixed feeding

A concerning finding is that less than a third of the participants in the current study were knowledgeable on mixed feeding. Furthermore, participants stated that mixed feeding is safe for HIV-positive mothers, and some disagreed that a baby can get HIV when mixed fed. Forty per cent of participants stated that infants can be given solids and breast milk from 3 months of age. The introduction of solids before 6 months to breastfed infants with HIV-positive mothers increases their chances of HIV transmission (Rollins et al. 2013). Human immunodeficiency virus-exposed infants who are not exclusively breastfed are susceptible to contracting HIV because the supplementary food given before 6 months damages the intestinal lining (Nankumbi & Muliira 2015). This may be the case with the participants in this study because they exhibited poor knowledge of mixed feeding and the dangers thereof.

Half of the participants (51%) in the current study indicated that mixed feeding includes giving the baby water, and 58.1% stated that a baby can get HIV when mixed-fed. Mixed feeding was the least known form of infant feeding among pregnant women in Cameroon (Sama et al. 2017). Only 34.1% (Dlamini & Mokoboto-Zwane 2019) of breastfeeding mothers in Eswatini knew they could infect their infants with HIV if they mix-fed. A lack of knowledge on mixed feeding can pose a danger for a potential mixed feeding episode.

In the current study, 51.6% of participants indicated that mixed feeding is safe when the mother is HIV-positive. This is true when ART is also included as ART can significantly reduce the risk of postnatal transmission when HIV-infected mothers either exclusively breastfeed or mixed feed (WHO 2016).

### Knowledge of replacement feeding

Less than half of the participants (49.0%) in the current study were knowledgeable about replacement feeding. Half of the participants (50.3%) reported that replacement feeding exposes the baby to possible allergens and intolerances. However, Ashipala et al. (2021) reported that 28.7% of HIV-positive mothers knew that replacement feeding puts the baby at risk of being ill and the baby could die if the formula is not prepared correctly. Poor knowledge of possible dangers associated with replacement feeding can make mothers choose it over breastfeeding (Ashipala et al. 2021). Most of the participants (79.4%) in a study carried out in Limpopo agreed that mothers who do not have sufficient milk should supplement with formula (Motadi et al. 2019).

### Attitude towards mixed feeding

More than a third of participants agreed and strongly agreed that mixed feeding is necessary to satisfy a baby, and a third indicated that mixed feeding is important for a baby to thrive. This attitude towards mixed feeding coupled with the participants’ poor knowledge of mixed feeding is a concern and requires interventions at a community level to better inform HIV-positive mothers on infant feeding.

### Attitudes on replacement feeding

More than half of the participants (58.7%) disagreed and strongly disagreed that they would stop breastfeeding if offered free formula at a hospital or clinic. Similarly, Ashipala et al. (2021) reported that 55.6% of HIV-positive mothers indicated that they would still continue to breastfeed even if they were offered free formula. For the participants to still breastfeed in situations where they are given free formula shows that they value the benefits of breastfeeding, and this could have a positive impact on the rates of breastfeeding at the Chief Albert Luthuli clinics in Mpumalanga.

### Infant feeding practices

Most of the participants (85.8%) in the current study reported that they had been advised to exclusively breastfeed for 6 months. More than half of the participants (61.3%) intended to exclusively breastfeed for 6 months and 29% intended to stop breastfeeding at 6 months. The proportion who intended to exclusively breastfeed for 6 months is greater than the 59.3% who intended to exclusively breastfeed found in a study carried out by Ashipala et al. (2021). The intention to exclusively breastfeed can lead to improved breastfeeding rates when mothers are motivated to breastfeed. However, a third of the participants in the current study intended to introduce solid foods before 6 months, thus increasing the risk of mixed feeding, the dangers of which have already been discussed.

### Strengths and limitations

This study focussed specifically on HIV-positive breastfeeding mothers as they are at greater risk than non-HIV if they practise improper feeding practices. However, the findings of this study are limited to the five clinics in the Chief Albert Luthuli sub-district and not all the clinics, thus limiting the generalisation of the results to a larger population.

### Recommendations

Based on the findings of this study, the following recommendations are presented related to practice, policy and research. For practice, it is recommended that ward-based outreach teams should also prioritise counselling and the support of HIV-positive breastfeeding mothers on infant feeding by doing regular visits, especially in the first 6 months, to support exclusive breastfeeding. Outreach services should also reach the HIV-positive mothers’ support systems and/or relatives to educate them on the benefits of accepting the HIV status and how this helps with adherence. Outreach services can be implemented in communities via radio talks, churches, taxi ranks, community halls and health care centres.

For policy, training and implementation of the policy should be strengthened in health care facilities to enable health care workers to educate mothers with up-to-date infant feeding recommendations. In addition, HIV-positive mothers should continue to be educated about all infant feeding options so that they may make informed choices about their infants’ feeding. Emphasis should be on education about and the promotion of breastfeeding, but mothers should be educated about the other infant feeding choices available to them.

Lastly, for research, more studies on the knowledge, attitudes and practices on infant feeding in HIV-positive mothers need to be conducted in the other sub-districts in the Gert Sibande district to substantiate the findings of this study. Qualitative studies would also give more value, as they would explore mothers’ experiences in greater detail.

## Conclusion

More than half of the participants were knowledgeable of exclusive breastfeeding. However, despite the appropriate knowledge of exclusive breastfeeding, the participants had many misconceptions which is a concern. Less than half of the participants were knowledgeable about exclusive breastfeeding in the context of HIV, mixed feeding and replacement feeding. Of all the infant feeding options, participants were least knowledgeable about mixed feeding.

Most of the participants in this study intended to exclusively breastfeed their infant for up to 6 months. However, there were some participants who intended to stop breastfeeding altogether at 6 months. This is because some believe that HIV-positive mothers cannot breastfeed after 6 months as this will put the infant at risk of mother-to-child transmission.
